# Faeces as a novel material to estimate lyssavirus prevalence in bat populations

**DOI:** 10.1111/zph.12672

**Published:** 2019-12-08

**Authors:** Lineke Begeman, Engbert A. Kooi, Erik van Weezep, Marco W. G. van de Bildt, Chantal B. E. M. Reusken, Peter H. C. Lina, Marion P. G. Koopmans, Judith M. A. van den Brand, Thijs Kuiken

**Affiliations:** ^1^ Department of Viroscience Erasmus University Medical Centre Rotterdam The Netherlands; ^2^ Wageningen Bioveterinary Research Lelystad The Netherlands; ^3^ Health and Youth Care Inspectorate National Authority for Containment Ministry of Health, Welfare and Sport Utrecht The Netherlands; ^4^ Centre for Infectious Disease Control‐RIVM Bilthoven The Netherlands; ^5^ Naturalis Biodiversity Center Leiden The Netherlands; ^6^ Department of Pathology, Veterinary Faculty University of Utrecht Utrecht The Netherlands

**Keywords:** Chiroptera, *Eptesicus serotinus*, faeces, lyssavirus, surveillance

## Abstract

Rabies is caused by infection with a lyssavirus. Bat rabies is of concern for both public health and bat conservation. The current method for lyssavirus prevalence studies in bat populations is by oral swabbing, which is invasive for the bats, dangerous for handlers, time‐consuming and expensive. In many situations, such sampling is not feasible, and hence, our understanding of epidemiology of bat rabies is limited. Faeces are usually easy to collect from bat colonies without disturbing the bats and thus could be a practical and feasible material for lyssavirus prevalence studies. To further explore this idea, we performed virological analysis on faecal pellets and oral swabs of seven serotine bats (*Eptesicus serotinus*) that were positive for European bat 1 lyssavirus in the brain. We also performed immunohistochemical and virological analyses on digestive tract samples of these bats to determine potential sources of lyssavirus in the faeces. We found that lyssavirus detection by RT‐qPCR was nearly as sensitive in faecal pellets (6/7 bats positive, 86%) as in oral swabs (7/7 bats positive, 100%). The likely source of lyssavirus in the faeces was virus excreted into the oral cavity from the salivary glands (5/6 bats positive by immunohistochemistry and RT‐qPCR) or tongue (3/4 bats positive by immunohistochemistry) and swallowed with saliva. Virus could not be isolated from any of the seven faecal pellets, suggesting the lyssavirus detected in faeces is not infectious. Lyssavirus detection in the majority of faecal pellets of infected bats shows that this novel material should be further explored for lyssavirus prevalence studies in bats.

**Impacts**

- People can acquire rabies by contact with rabid bats. This makes bat rabies of concern for both public health and bat conservation.
- There is limited knowledge on the epidemiology of bat rabies hampering the application of preventive measures. Therefore, we should improve our strategies to investigate free‐ranging bat populations for rabies prevalence.
- Our finding of lyssavirus RNA in faecal pellets of six out of seven confirmed rabid bats suggests testing faeces should be further explored as a strategy for epidemiologic studies.

## 1 INTRODUCTION

Rabies is a fatal neurologic disease caused by an infection with a lyssavirus. People usually acquire the infection from bites by infected carnivores or bats. The pathogenesis is similar in all species, including bats. Typically, virus enters a new host via a bite from an infected host and infects nerves in the area of the bite. Once in the nervous system, the virus spreads to the brain, and from there reaches nerves of the salivary glands and the tongue. Once it is in these organs, virus is excreted into the oral cavity, from where it can be transmitted to the next host (Begeman et al., 2018).

Lyssaviruses circulate in bats worldwide, and new lyssavirus species are identified regularly (Aréchiga Ceballos et al., 2013; Banyard, Evans, Rong Luo, & Fooks, 2014; Nokireki, Tammiranta, Kokkonen, Kantala, & Gadd, 2018). Virus prevalence estimation and testing for presence or absence of current infection in bat populations is necessary for mitigation of public health risks as well as for bat conservation. Two methods are used. One is to catch bats from a population, take oral swabs and test them for the presence of virus (Schatz, Ohlendorf, et al., 2014). This requires acquisition of permits, trained and vaccinated personnel and is both unpractical and invasive. The other is to test brains of bats found ill or dead, and this subset of bats is not representative of the bat population as a whole (Schatz, Freuling, et al., 2014). Testing for lyssavirus specific antibodies in bat colonies is another strategy to understand lyssavirus epidemiology (Robardet et al., 2017), but does not distinguish between current and past infection or exposure, and therefore does not give any information about current virus prevalence. Thus, there is a need for an alternative method.

Faeces sampling has shown to be effective to determine the prevalence of other viral infections in bat populations (Drexler et al., 2011). So far, faeces have not been tested as a material for lyssavirus prevalence studies. It is perhaps counterintuitive to do so because lyssaviruses target the nervous system and not the digestive system. Still, one study showed that rabies virus RNA can be detected in faeces of infected bats (10 of 25 [40%] positive) (Allendorf et al., 2012). Therefore, we performed a pilot study to evaluate faeces as a material for lyssavirus prevalence studies.

## 2 MATERIALS AND METHODS

We received bat carcasses from already existing collections of bat rehabilitators who gave us their consent to use the carcasses for this investigation. These bats either had been found dead or had been euthanized by the bat rehabilitators because of bad prognosis for recovery. The bat carcasses were transported to our facility and investigated under permit FF/75A/2015/036 from the Dutch Ministry of Economic Affairs. On 21 serotine bat carcasses, *Eptesicus serotinus* (Schreber 1771), that died in the Netherlands between December 2016 and December 2018, extensive autopsies were performed. All bats were tested for European bat lyssavirus (EBLV‐1) RNA in the brain. Eight of these 21 tested positive, and of seven of the eight bats, a faecal sample was available for further testing, and these were thus selected for our study. One serotine bat from the same series, which tested negative for EBLV‐1 RNA in the brain, and for which a faeces sample was available, was selected as negative control. Autopsies of these eight bats took place after storage of the carcasses at −20°C variably up to 17 months. Faecal pellets were taken from the rectum of all eight bats at autopsy, with one exception. The exception was an EBLV‐1‐positive bat whose rectum was empty at autopsy. Instead, faecal pellets collected at 3 and 2 days before death of this bat from its cage in a rehabilitation centre were used. In addition to faecal pellets, samples collected at autopsy included oral swabs and tissue samples of brain, salivary gland and intestine. Faecal pellets and oral swabs were stored in virus transport medium at −80°C directly after sampling. Tissue samples of brain, salivary gland and intestine were stored −80°C. These samples remained at −80°C for 14 months before testing took place. Duplicate tissue samples of salivary gland and intestine, as well as samples of tongue, were fixed in 10% neutral‐buffered formalin, embedded in paraffin wax and cut in 4‐μm‐thick sections within 3 weeks after autopsy.

We tested faecal pellets, oral swabs and tissue samples of all eight bats for lyssavirus RNA by use of RT‐qPCR according to the protocol of Schatz (2014) with minor modifications. The resulting quantification cycle (*C*_q_) values were inversely correlated with the amount of specific RNA that was detected in the original sample. On RT‐qPCR positive faecal samples RT‐PCR was performed, and products were sequenced according to the protocol of Heaton et al., (1997) to ensure the amplicon's specificity. We compared the sensitivity of lyssavirus detection by RT‐qPCR in faeces and oral swabs. We also tested faecal pellets for infectious virus by virus culture (Webster & Casey, 1996) and used the brains of the bats as positive controls. We evaluated potential sources of lyssavirus RNA in faeces by comparing the *C*_q_ values in faecal pellets with those in the salivary glands and intestine, and by examining tongue, salivary gland and intestine for lyssavirus antigen by immunohistochemistry (IHC) (Suu‐Ire et al., 2018).

## 3 RESULTS

**Figure 1:**
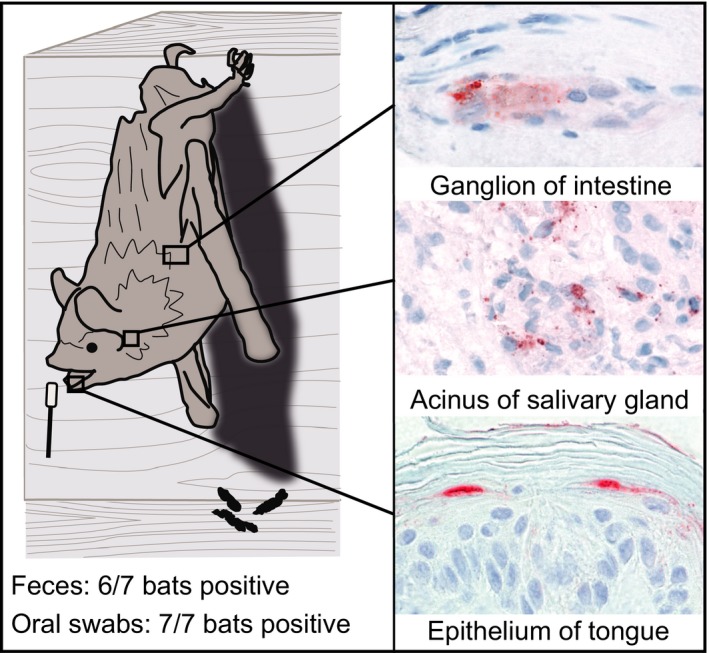
Results of testing faeces of seven serotine bats naturally infected with European bat lyssavirus 1, as a novel material for lyssavirus prevalence studies. Left side: Faecal samples (6/7 bats) tested nearly as sensitive as oral swabs (7/7 bats) for the detection of lyssavirus RNA by RT‐qPCR. Right side: Lyssavirus antigen expression (red) in tissues of these bats show potential source of virus. Most likely source of virus was considered to be salivary gland (middle panel, showing positive epithelial cells within an acinus) and/or tongue (bottom panel, showing positive epithelial cells on surface of tongue). Intestine (top panel, showing positive neurons in myenteric ganglion) was considered to be a less likely source because there is no known route of excretion of lyssavirus from intestinal wall to intestinal lumen. Original magnification of all panels 100× objective [Colour figure can be viewed at wileyonlinelibrary.com]

Detection of lyssavirus infection was nearly as sensitive in faecal pellet samples (6/7 bats [86%] positive by RT‐qPCR) as in oral swabs (7/7 [100%]; Figure 1). The mean *C*_q_ value of the oral swabs was 24, with a range of 19–27, while the mean *C*_q_ value of faecal pellets was only two higher, with a mean of 26 and a range of 21–29. This suggests that although viral loads in faeces are lower than in oral swabs of any of these seven bats, the differences are relatively small. The amplicon's specificity could be confirmed in two of six RT‐qPCR positive faecal samples by sequencing the entire *N* gene (1,611 nucleotides) of the RT‐PCR product. One of the six RT‐qPCR positive faecal pellets was the sample taken from a live bat that was being cared for at a rehabilitation centre, and that died 4 days later of rabies. This shows it is possible to detect virus in faeces of live bats. Despite detection of lyssavirus RNA, virus could not be cultured from faecal pellets of any of the seven bats, suggesting it did not contain infectious lyssavirus. In contrast, lyssavirus was cultured from brains of five (71.4%) of the seven bats, indicating storage conditions still allowed successful virus culture.

The evidence of lyssavirus infection in salivary gland (5/6 [83.3%] bats positive by RT‐qPCR, mean *C*_q_ 19, range 15–28; 5/6 [83.3%] positive by IHC) and/or tongue (4/5 [80%] positive by IHC; for two bats, tongue samples were not available, RT‐qPCR not done) suggested that these tissues were the likely sources of lyssavirus RNA in the six positive faeces samples. It cannot be ruled out that lyssavirus originated from the intestinal wall (5/6, mean *C*_q_ 23, range 17–29; 5/6 positive by IHC; Figure 1). However, there is no known route of excretion of lyssavirus from intestinal wall to intestinal lumen.

## 4 DISCUSSION

The conclusion from this pilot study is that faeces are a suitable material for the detection of EBLV‐1 in bats around the time of death. The likely sources of lyssavirus in the faeces are from salivary glands, tongue or both, from which virus is excreted into the oral cavity and subsequently swallowed with saliva. This is based on our knowledge that these organs are sources of lyssavirus excretion in bats (Begeman et al., 2018) and is supported by the RT‐qPCR and IHC results of salivary gland and tongue samples in this study.

Whether faeces can be used for lyssavirus prevalence studies of free‐living bat populations remains to be determined. For this use, lyssavirus needs to be detectable in faeces of infected bats not only around the time of death but also at the preclinical stage, when the bats are apparently healthy. Results from experimental infections provide evidence of preclinical lyssavirus excretion in bats. In bats of different species inoculated with a variety of lyssaviruses, infected animals excrete lyssavirus in saliva, the likely source of RNA in our faeces samples, for several days to weeks prior to the occurrence of death, and before they show clinical signs (Table 1). Thus, we also expect faeces of free‐ranging, lyssavirus‐infected bats to contain detectable lyssavirus RNA at the preclinical stage.

**Table 1:** Literature review presenting evidence for lyssavirus excretion prior to death and prior to clinical signs in experimentally inoculated bats

Lyssavirus species	Bat species	No. of successfully infected bats^a^	Maximum day of virus detection in oral swabs prior to death for each bat in which it was detected.	Excretion detected prior to clinical signs	References
European bat 1	*Eptesicus fuscus*	15	2; 7; 11; 14; 37	N.r.	Franka et al. (2008)
European bat 2	*Myotis daubentonii*	1	4	Yes	Johnson et al. (2008)
Khujand	*Eptesicus fuscus*	3	1; 4	Yes	Hughes et al. (2006)
Rabies	*Eptesicus fuscus*	13	2; 7	Yes	Davis, Jarvis, Pouliott, and Rudd (2013)
Rabies	*Eptesicus fuscus*	16	1; 1	N.r.	Jackson et al. (2008)
Rabies	*Eptesicus fuscus*	6	4;13	Yes	Davis, Gordy, and Bowen, (2013)
Rabies	*Myotis lucifugus*	2	13; 18	Yes	Davis, Jarvis, Pouliott, Morgan, and Rudd (2013)
Rabies	*Myotis lucifugus*	1	14	N.r.	Stamm, Kissling, and Eidson (1956)
Rabies	*Desmodus rotundus*	26	8; 9; 9; 10; 10; 11; 11; 12; 12; 12, 13	Yes	Moreno and Baer (1980)
Rabies	*Tadarida brasiliensis*	24	3; 5; 5; 6; 7; 7; 10; 11; 11; 12; 14; 14; 15; 15; 15; 16; 16; 20	Yes	Baer and Bales (1967)

Abbreviation: N.r., not recorded in the study.

a The number of bats in the experiments for which rabies was confirmed by lyssavirus detection in the brain.

Our study implies that faeces should be further explored as a material for prevalence studies of lyssavirus infections in bats. It should be taken into account that the prevalence of lyssavirus infection in reservoir populations during non‐epidemic periods is expected to be low (0.7%–3%) (Mørk & Prestrud, 2004; Schatz, Ohlendorf, et al., 2014). For example, if the expected prevalence in a population of 200 bats is 2%, a sample size of 105 is required to state absence or presence of lyssavirus infection (95% confidence interval, *p* < .05). For a population of 1,000 bats or more, required sample size is 148. Because faeces are often so easy to collect under bat roosts, these are feasible sample numbers (Thrusfield, 1986).

We realize that further validation is needed before it can be determined whether faecal pellets can be used for lyssavirus prevalence studies in free‐living bat populations. Questions remaining include how many days prior to disease or death lyssavirus RNA can be detected in faeces of infected bats, and how long lyssavirus RNA can be detected in bat faeces after defaecation, and how to relate the number of sampled faecal pellets at a roosting site to the number of bats present at that site. However, we wish to share our idea and these preliminary results so that other researchers investigating lyssavirus infections in bats have the opportunity to explore this sampling strategy. The circulation of lyssaviruses in bats is a concern for both public health and bat conservation (Banyard et al., 2014; Begeman et al., 2018). With the proposed novel sampling strategy, we hope to contribute to an increased understanding of the epidemiology of rabies in bats.

## CONFLICT OF INTEREST

The authors declare no conflict of interest.
